# Advances and Classification of Cyclodextrin-Based Polymers for Food-Related Issues

**DOI:** 10.3390/polym13234226

**Published:** 2021-12-02

**Authors:** Adrián Matencio, Alberto Rubin Pedrazzo, Alessandro Difalco, Silvia Navarro-Orcajada, Yousef Khazeai Monfared, Irene Conesa, Azam Rezayat, José Manuel López-Nicolás, Francesco Trotta

**Affiliations:** 1 Department of Chemistry, University of Turin, Via P. Giuria 7, 10125 Torino, Italy; alberto.rubinpedrazzo@unito.it (A.R.P.); alessandro.difalco@unito.it (A.D.); yousef.khazaeimonfared@unito.it (Y.K.M.); azamrezayat1981@gmail.com (A.R.); 2 Department of Biochemistry and Molecular Biology A, Biology Teaching Unit, Facultad de Veterinaria, Regional Campus of International Excellence “Campus Mare Nostrum”, Universidad de Murcia, 30100 Murcia, Spain; silvia.navarro6@um.es (S.N.-O.); irene.conesav@um.es (I.C.); josemln@um.es (J.M.L.-N.); 3 Department of Chemistry, Faculty of Science, Lorestan University, Khorramabad 6813833946, Iran

**Keywords:** cyclodextrins, materials, polymers, food industry, review

## Abstract

Cyclodextrins (CDs) are a good alternative to reduce or enhance different biomolecule characteristics and have demonstrated great results in food science. However, CDs present intrinsic limitations that can be solved by derivative synthesis. This review represents a survey of the state of the art of CD-based materials and their uses in food science. A deep review of the structure is carried out and different groups for ordination are suggested. After that, different applications such as cholesterol complexation or its use as sensors are reviewed. The derivatives show novel and promising activities for the industry. A critical perspective of the materials suggests that they might not present toxicity, although more studies are required. These points suggest that the research in this field will be increased in the following years.

## 1. Introduction

Society is increasingly demanding healthier food products, prompting research on novel ways to improve them. This demand could be satisfied by fortifying foods with bioactive compounds (molecules that have demonstrated effects as antioxidants or anticancer activities, among others), removing undesirable substances, preventing microbial contamination or changing organoleptic properties.

**Figure 1:**
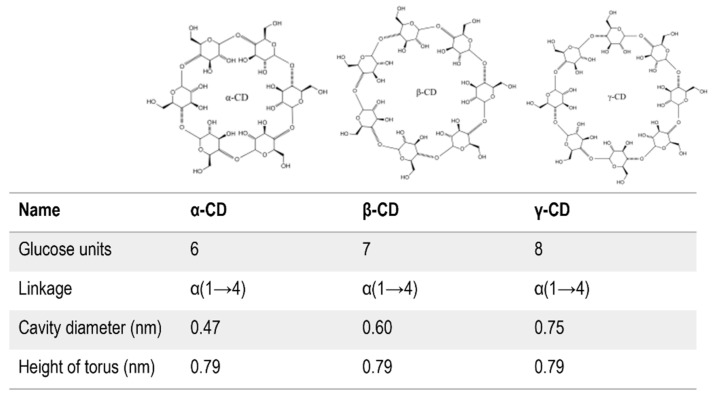
Most common CDs in food science and their characteristics.

One possible method of managing these strategies is the use of cyclodextrins (CDs, Figure 1). CDs are truncated cone-shaped oligosaccharides made up of α-(1,4) linked glucose units, obtained from the degradation of starch by the enzyme cyclodextrin glucosyltransferase (CGTAse). The most common in the food industry present six, seven and eight glucose units, (called α-, β- and γ-CD, respectively) [1]. These natural CDs possess the E numbers E-457, E-459 and E-458 for α-, β- and γ-CD, respectively, as members of the food additives list and have two GRAS statuses. Although both inorganic and organic salts and neutral molecules can form complexes with CDs, they are more generally used to complex poorly soluble drugs or bioactive compounds (BaCs), creating “inclusion complexes” [2,3]. In solution, the equilibrium between free and complexed BaCs is dynamic, meaning that when the solution is diluted or another BaC is added, the first BaC is easily released.

In general, the inclusion complex formation improves the apparent solubility of the drug, which increases its final concentration, even affecting the BaC structure and possibly its bioactivities [4,5,6,7,8,9]. However, other effects such as increased stability, controlled release or chiral separation occur when the inclusion complex is formed [10]. Finally, their uses as active agents (without a guess) are being also studied for different diseases or applications [3,11].

In 2020, our research group published a comprehensive review about the applications of CDs in food science [2]. Nevertheless, there was little evidence of the use of CD-based polymers focused on the food industry. In this new review, collaborative work with CD chemical specialists has been done to prepare a critical viewpoint of the promising possibilities of these derivatives in the field.

## 2. Classification of CD-Based Polymers

The continuous research on efficient, effective and cheaper materials has moved the interest towards natural polymers, such as polysaccharides, starch, chitin [12,13,14,15,16,17,18] and, of course, cyclodextrins and derivatives.

**Figure 2:**
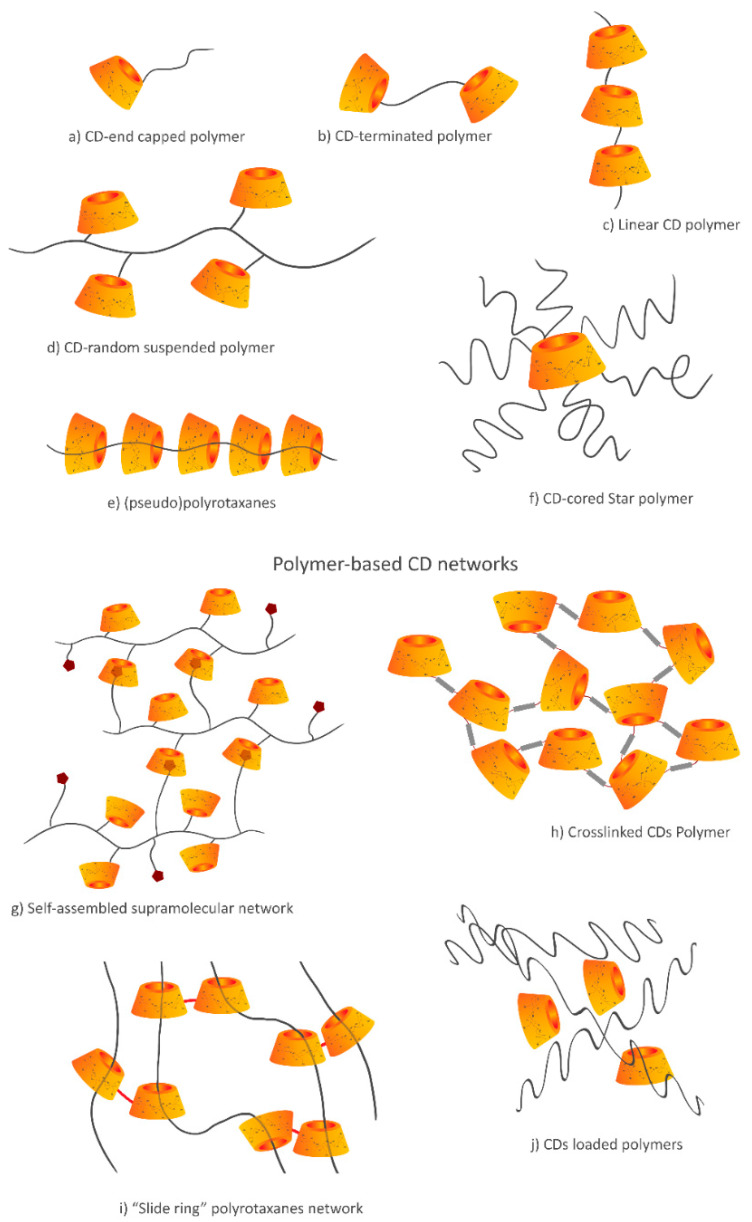
Representations of most common CD-based polymers and materials. (**a**) CD-end capped polymer (**b**) CD-terminated polymer (**c**) Linear CD polymer (**d**) CD-random suspended polymer (**e**) (pseudo)Polyrotaxanes (**f**) CD-cored star polymer. CD based networks: (**g**) Self-assembled supramolecular network (**h**) Crosslinked CDs polymer (**i**) “Slide ring” polyrotaxanes network (**j**) CDs loaded polymers.

**Scheme 1:**
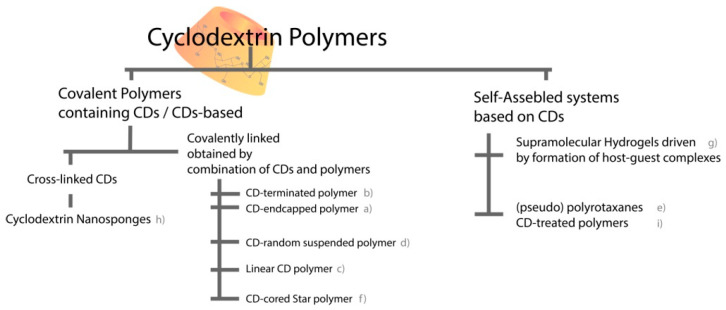
Detailed overview of CD-based polymers.

In previous reviews [19,20,21,22], CD polymers were categorized by structures, according to differences in shape and CD organization. Scheme 1 and Figure 2 summarize and represent the most common examples of CD polymers with the intention of giving an overview of the possible polymers structures, only focusing on the CD polymers reported to be used in food-related applications. Therefore, a detailed description of the many syntheses and applications is beyond the scope of the present work, but an overview of the many possible CD-based structures is, in our opinion, necessary.

Bailey et al. in 1999 defined a material as “low cost” if it requires little processing and if is abundant in nature (or a by-product or waste of other processes) [14]. Polysaccharides are, consequently, remarkably interesting from the economic point of view: they are a renewable and biodegradable resource with good physicochemical characteristics, chemical stability and high reactivity thanks to the presence of chemical reactive hydroxyl groups. 

Focusing on CD, the literature contains several methods to achieve an enhancement of the well-known and already described complexation ability of CDs. The first approach is the derivatization of CDs, which permits significant results to be obtained [23,24]. Another strategy, somewhat related to derivatization, since a chemical reaction is involved, concerns the polymerization of CDs to obtain either soluble or insoluble polymers [22,25].

There are many advantages that derive from the polymerization of cyclodextrins [22,26,27,28]: one of the most interesting is the possibility to form complexes with a wider variety of molecules [25], being able to exploit both the slightly hydrophobic cavity of the cyclodextrins (and their ability to form inclusion complexes) and the hydrophilic external part. Moreover, the peculiar structure of CDs enables the facile synthesis of polymers characterized by different architectures, very difficult to obtain by other means [22]. Using CDs as building blocks, it is possible to synthesize linear or star polymers [19,20,22], polyrotaxanes (PRs) [29,30] or supramolecular polymers [28,31,32,33]. Thus, the possibility to form supramolecular host–guest interactions allows the possibility to obtain stimuli-responsive materials. The most important advantage to stress is that CD-based biopolymers exhibit good biocompatibility and are easy to process [22].

In 2009, Van de Manakker et al. [34] proposed a division into two categories of CD-based polymers, essentially based on the presence or absence of covalent bonds. In two recent reviews, as an alternative, Tian et al. [19,20], reprising what was extensively described by Yao in 2019 [22], proposed a categorization more focused on the “final structure” and on the role of the cyclodextrin in the construction of the polymer. Similarly, Liu et al., in a recent and comprehensive review [35], divided cyclodextrin polymers into four categories, according to their structures (CD-based polyrotaxanes, grafted CD polymers, crosslinked CD polymers and CD-based star polymers). This division, in our opinion, takes into account the final structure but does not immediately clarify the presence of bonds or the presence of supramolecular forces. Since cyclodextrin polymers are strictly employed in quite different applications according to their (in)solubility, it is, in our opinion, essential to make this distinction first.

In Scheme 1 and Figure 2, we extend these categorizations, reporting the type of polymer and some examples of simplified structures. The first categorization is the absence or presence of covalent bonds between CDs and an external polymer and between each CD with linkers (such as for crosslinked CD polymers). It is then possible to make further subdivisions essentially on the “position” of the cyclodextrins in the system structure (as the core of different polymer chains, as pendants, etc.). 

Based on the above, it is possible to deduce that the synthesis of cyclodextrin polymers strictly depends on the final structure and on the application as well. 

Focusing on CD-based covalent polymers, Seidi et al. recently extensively described many different syntheses [36] which however refer to only two distinct approaches: the direct polymerization or copolymerization of pristine cyclodextrins (or cyclodextrin derivatives) with various monomers or linkers including isocyanates, epoxides, carboxylic acids, anhydrides, acrylamides and halogenated aromatic compounds and the postfunctionalization of other polymers with CDs or CD derivatives. 

The first approach is usually a direct polycondensation of CD units with other multifunctional agents. The orientation of the hydroxyl groups toward the exterior side of CDs allows them to act as polyfunctional monomers easily participating in a huge variety of polymerizations, including ring-opening polymerization with epoxides [19,37,38], polyesterifications with carboxylic acids or related derivatives [39,40], formation of polyurethane with isocyanates, and nucleophilic substitution with halogenated aromatic compounds [25,41,42]. 

The most common and widely reported synthetic method is cross-linking the CDs with epichlorohydrin (EPH; 1-chloro-2,3-epoxy propane) [43,44,45]. This first strategy easily permits obtaining crosslinked and hyper-crosslinked systems, and the prefunctionalization of CDs is not generally required [41,45].

The limitations for direct copolymerization of CD units are essentially due to the fact that native CDs only contain hydroxyl groups. Moreover, direct utilization of CDs in polymerizations usually leads to the hyperbranched or crosslinked polymers [46], whereas by using functionalized derivatives of CDs, it is possible to produce linear CD polymers and to better control the final structure [47]. Many examples can be found in the literature [19,36,48,49,50]. A common, widely reported method, for example, involves the preparation of various amino derivatives of β-CD, which can act as bifunctional monomers and are used for polymerization with diimidates [51,52].

For what concerns different organizations, as already said, the synthesis is closely related to final structure. As an example, in the case of polymers bearing CD unit pendant groups (Figure 2d), there are two methods for obtaining CD “decorated” polymers. The first one involves the synthesis of vinyl derivatives of CDs that then can be incorporated in a radical copolymerization as monomers [34,53]. The other possible method is based on the conjugation of CD derivatives as side chains of a prefabricated or natural polymer [54,55].

As reported in Scheme 1, the other possibility of organization of CD polymers is based on the self-assembling of CDs and polymer via the formation of host–guest complexes (Scheme 1 and Figure 2g) or polyrotaxanes (Scheme 1 and Figure 2e) or more complex and specific structures [56,57,58]. However, it is necessary to underline that these systems are not necessarily free of covalent bonds; indeed, many times we can find examples of complex structures based on both supramolecular forces and covalent bonds [29,59].

For completeness, examples are reported in this section, but it is necessary to take into account that in most cases the cavities of the cyclodextrins are occupied by the polymer itself or by molecules which lead to the formation of lattices or hydrogels, thus preventing the possibility of forming further inclusion complexes in the CD cavity, crucial for most food-related applications [19,60,61].

## 3. Applications of Cyclodextrin-Based Materials and Polymers in Food Science

**Scheme 2:**
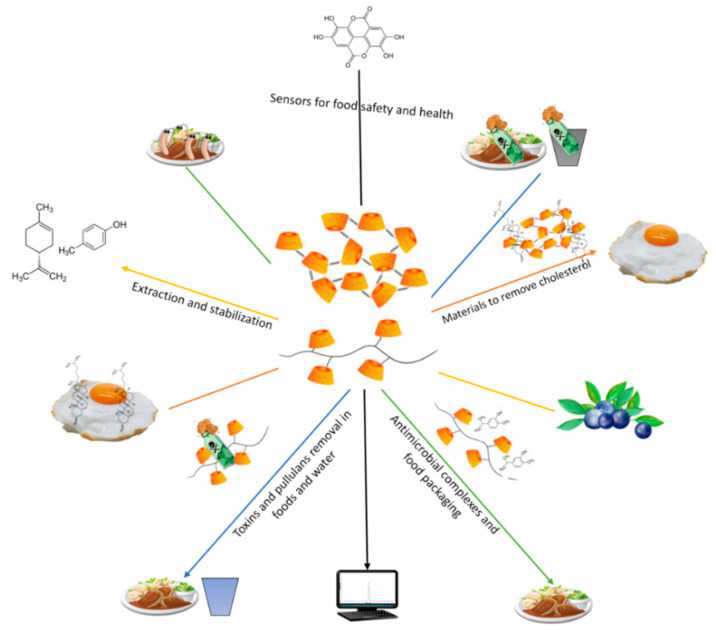
Representation of the most representative current applications.

As mentioned above, the need for an overview of the application of CD-based materials and polymers in food science has led us to provide some guidance for the community involved. We cannot dismiss the possibility of CD-based materials being used to complex bioactive compounds and enhance their bioactivities. However, this aspect is not reviewed due to the high number of biomolecule complexes, e.g., kynurenic acid or stilbenes [62,63,64]. A selection of the most interesting applications are represented in Scheme 2 and summarized in Table 1.

### 3.1. An Overview of the Use of Cyclodextrin-Based Materials to Complex Cholesterol 

The complexation of cholesterol by CDs is a powerful strategy not only in food science [2] but also in pharmaceutical applications such as Niemann–Pick type C, atherosclerosis or antiviral potential [65,66,67,68]. Novel strategies based on CDs in food science have been developed to improve the results of CD blocks in food science, above all for milk and dairy products. In an interesting paper, beads of chitosan with β-CD immobilized by cross-linking with 1,6-hexamethylene diisocyanate (Ch-βCD) were used to remove cholesterol from egg yolk [69]; the cholesterol adsorption capacity of this material was 0.33 g cholesterol/g adsorbent, and it was able to adsorb the 92% of the cholesterol from a 30-fold diluted yolk solution at 25 °C in 2 h using 1% (*w*/*v*) Ch-BC. In addition, after 12 reuses, 84% and 90% of the adsorption and desorption capacities were retained. Other crosslinked CDs have been proposed to complex cholesterol; for example, with an adipic acid–β-CD crosslinked material, over 90% of cholesterol was removed at a 1:1 ratio of egg yolk to water with 20% of crosslinked β-CD added and mixing for 30 min at 40 °C [70], representing a good improvement compared to the previous example. On the other hand, the use of epichlorohydrin derivatives was proposed to recover and recycle the CD polymer, but with a decrease in the removal of cholesterol (92.0% free β-CD against 82.6%) [71]. 

The use of the immobilization of β-CD in glass surfaces was also proposed to easily recover the material [72]. However, the authors found a lower yield than using free β-CD. Cellulose nanocrystals have been recently used to create a crosslinked polymer with β-CD in presence of glutaraldehyde as a linker to create a CD-modified poly (HEMA-GMA-g-CNC) column. Using a continuous system, it was able to complex around 99% of cholesterol and low-density lipoprotein with agreement between experimental data and mathematical models, being an interesting future alternative [73].

Although there are several examples of CD-based materials for cholesterol complexation patented [68,74,75,76], the use of β-CD without modifications continues to be the most used strategy in food science [2,77,78].

**Table 1:** Summary of the applications and classification of more relevant studies.

Application	Material Classification	Type of Material	Biomolecule	Effect	Reference
Cholesterol extraction	CD random suspended polymer	Chitosan with immobilized β-CD	Cholesterol	Cholesterol extraction	[69]
Cholesterol extraction	Crosslinked CD polymer	Adipic acid with β-CD	Cholesterol	Cholesterol extraction	[70]
Cholesterol extraction	Crosslinked CD polymer	Epichlorohydrin and β-CD	Cholesterol	Cholesterol extraction	[71]
Cholesterol extraction	Crosslinked CD polymer	Cellulose and β-CD with glutaraldehyde	Cholesterol	Cholesterol extraction	[73]
Contaminants	Crosslinked CD polymer	Active carbon CD	Methylene blue	Contaminant removing	[79]
Contaminants	Crosslinked CD polymer	Cellulose nanofibrils/β-CD	Microcystin-LR/methylene blue	Contaminant removing	[80]
Contaminants	Crosslinked CD polymer	Cyclodextrin-based nanosponges (CD-NSs)	2-MIB	Contaminant removing	[81]
Contaminants	Crosslinked CD polymer	CD-NSs	Ciprofloxacin	Contaminant removing	[82]
Contaminants	Crosslinked CD polymer	CDs containing amino and amido groups	PFAS	Contaminant removing	[83]
Contaminants	CD end-capped polymer	Amphiphilic CDs in PFS membrane	Endocrine-disrupting plasticizer	Contaminant removing	[84]
Contaminants	Crosslinked CD polymer	Mesoporous β-CD polymers	Heavy metals	Contaminant removing	[85]
Contaminants	Self-assembled supramolecular network	Insoluble β-CD bead polymers	ZEN	Contaminant removing	[86]
Contaminants	Self-assembled supramolecular network	Insoluble β-CD beads polymers	AOH	Contaminant removing	[87]
Contaminants	Crosslinked CD polymer	CD-NSs	Indole	Contaminant removing	[88]
Extraction	Crosslinked CD polymer	CDs crosslinked with epichlorohydrin, hexamethylene diisocyanate and phenyl isocyanate	Naringin and limonin	Extraction of undesirable molecules	[89]
Extraction	Crosslinked CD polymer	CM-HP-β-CDCP-MNPs	Rutin	Extraction of bioactive compound	[90]
Food packaging	CD-loaded polymer	β-CD + low-density polyethylene	Carvacrol/trans-cinnamaldehyde	Antimicrobial	[91]
Food packaging	Crosslinked CD polymer	β-CD + chitosan	Carvacrol/trans-cinnamaldehyde	Antimicrobial	[92]
Food packaging	Crosslinked CD polymer	β-CD + microfibrillated cellulose	Carvacrol	Antimicrobial	[93]
Food packaging	CD-loaded polymer	β-CD + halloysite nanotubes	Carvacrol + cinnamon + oregano essential oils	Antimicrobial	[94]
Food packaging	Crosslinked CD polymer	HPβ-CD + TEMPO-oxidized cellulose nanocrystals	Carvacrol/curcumin	Antimicrobial	[95]
Food packaging	-	Titanium dioxide nanoparticles with α-CD and β-CD	Sorbic acid/benzoic acid	Antimicrobial	[96]
Food packaging	Crosslinked CD polymer	α-NS, β-NS and HPβ-NS	Coriander essential oil	Antimicrobial	[97]
Food packaging	Crosslinked CD polymer	α-NS, β-NS and HPβ-NS	Cinnamon essential oil	Antimicrobial	[98]
Food packaging	CD-loaded polymer	β-CD + chitosan edible coating	trans-Cinnamaldehyde	Antimicrobial	[99]
Food packaging	CD-loaded polymer	β-CD + sodium alginate edible coating	trans-Cinnamaldehyde	Antimicrobial	[100]
Food packaging	CD-loaded polymer	α-CD + polystyrene	Ethylene	Ripening control	[101]
Food packaging	CD-loaded polymer	α-CD + polystyrene (electrospun nanofibers)	1-Methylcyclopropene	Ripening control	[102]
Food packaging	-	γ-CD + metal–organic frameworks	Hexanal	Ripening control	[103]
Food packaging	CD-loaded polymer	CD + polyvinyl chloride		Capturing contaminants	[104]
Food packaging	Self-assembled supramolecular network	β-CD + zein	Cholesterol	Reduction of cholesterol	[105]
Food packaging	CD-loaded polymer	Triacetyl β-CD + low-density polyethylene (electrospun nanofibers)	Sulfur off-flavors	Fragrant	[106]
Food packaging	CD-loaded polymer	β-CD + soy protein + polyethylene oxide (electrospun nanofibers)	Allyl isothiocyanate	Antimicrobial	[107]
Food packaging	CD-loaded polymer	β-CD + polylactic acid (electrospun nanofibers)	trans-Cinnamaldehyde	Antimicrobial	[108]
Food packaging	CD-loaded polymer	β-CD + zein (electrospun nanofibers)	Eucalyptus essential oil	Antimicrobial	[109]
Food packaging	CD-loaded polymer	β-CD + polyvinyl alcohol + lysozyme (electrospun nanofibers)	Cinnamon essential oil	Antimicrobial	[110]
Food packaging	CD-loaded polymer	β-CD + chitosan + polyvinyl alcohol + lysozyme (electrospun nanofibers)	Cinnamon essential oil	Antimicrobial	[111]
Food packaging	CD-loaded polymer	γ-CD + zein (electrospun nanofibers)	Quercetin	Antioxidant	[112]
Food packaging	CD-loaded polymer	HPβ-CD + gliadin (electrospun nanofibers)	Ferulic acid	Antioxidant	[113]
Food packaging	CD-loaded polymer	γ-CD + polyvinyl alcohol (electrospun nanofibers)	Geraniol	Antimicrobial, antioxidant and fragrant	[114]
Food packaging	CD-loaded polymer	β-CD + pullulan (electrospun nanofibers)	D-Limonene	Antimicrobial and fragrant	[115]
Food packaging	CD-loaded polymer	β-CD + chitosan	2-Phenyl ethanol	Antimicrobial and fragrant	[116]
Detection of BPA	Crosslinked CD polymer	β-CD polymer film	Bisphenol A (BPA)	Enhancement of fluorescence intensity	[117]
Detection of NPN	(Molecularly imprinted) crosslinked CD polymer	β-CD polymer particles	N-Phenyl-1-naphtylamine	Enhancement of fluorescence intensity	[118]
Detection of AdCA	CD-suspended polymer	Poly(phenylene-ethylene) β-CD appended	1-Adamantanecarboxylic acid	Shift of fluorescence peak	[119]
Detection of 1-phenylethylamine	CD-suspended polymer	(Stereoregular) polyphenylacetylene β-CD appended	1-Phenylethylamine	Shift of fluorescence peak	[120]
Detection of chlorinated phenols	Linear CD polymer	Azo-dye modified CD-polyoxyethylene	Chlorinated phenols	Quenching of fluorescence intensity	[121]
Detection of nitroarenes	Crosslinked CD polymer	CD-functionalized polyarene	Trinitrophenol, nitrobenzene	Quenching of fluorescence intensity	[122]
Detection of captopril	Crosslinked CD polymer	β-CD functionalized poly-TFT	Captopril	Activation of fluorescence	[123]
Detection of benzene	Crosslinked CD polymer	CD/maleic acid copolymer	Benzene	Quartz crystal microbalance sensing	[124]

### 3.2. Cyclodextrin-Based Materials for Contaminant or Toxin Removal

#### 3.2.1. Water

Granular activated carbon, one of the most used materials, has limitations for the adsorption of organic molecules in low concentrations [125]. From various materials, CDs and their derivatized materials present a good alternative for removing contaminants.

The promising capacities of CD-based aerogels should be discussed. The porous nature of aerogel, in combination with the capacities of CDs to complex pollutants, suggests several applications. Several cross-linkers such as hexamethylene diisocyanate or triphenylmethane are used to generate gelled CDs for aerogel preparation [126]. Using methylene blue as sample, active carbon CD aerogels were synthesized and tested with an adsorption capacity of 208.33 mg/g at 323 K involving two processes: external surface adsorption and intraparticle diffusion [79]. In another interesting study, crosslinked cellulose nanofibrils/β-CDs were synthesized with EPH for studying water pollutant complexation after aerosol preparation; using microcystin-LR as an example, they found an adsorption capacity of 0.078 mg/g when the concentration was 0.8 μg/mL [80].

Mhlanga et al. exploited a new class of polyurethane cyclodextrin-based nanosponges (CD-NSs) for removing model contaminants such as 2-methylisoborneol (2-MIB) [81] from water [127,128]. The results demonstrated that nanosponges have a higher affinity than granular activated carbon (CAC) to remove and absorb organic pollutants, especially 2-MIB. Moreover, nanosponges have been found to remove other molecules such as heavy metals or drugs such as ciprofloxacin efficiently [39,82].

The presence of per- and polyfluoroalkyl substances (PFAS) can be managed with β-cyclodextrin polymers containing amino and amido groups. In a study, Yang et al. compared different β-CD polymers. The results demonstrated that the affinity of the polymers containing amine groups for removal of anionic PFAS was higher than that of amido polymers [83].

In another study, Chio et al. tried to remove endocrine-disrupting plasticizer from water: Firstly, they synthesized β-cyclodextrin modified with fatty acid for the preparation of amphiphilic β-CDs. Then, they incorporated the amphiphilic β-CDs into a polysulfone (PSF) membrane to obtain a PSF/CD membrane through an inverse phase procedure. Two different membrane types were tested: flat sheet and hollow fiber membrane. The performance of these membranes was considered: results demonstrated that the efficiency of PSF/CD membranes in the elimination of endocrine-disrupting plasticizer from water increased remarkably with the amount of β-CD and that these membranes can be applied as a commercially available and eco-protective water treatment system [84]. Mesoporous β-CD polymers with crosslinked rigid aromatic structures were used as absorbents for the removal of heavy metals such as Pb, Cu and Cd from water. The results demonstrated that the affinity of the β-CD polymer for adsorption of the three heavy metals was Pb > Cu > Cd [85]. 

#### 3.2.2. Body and Food Matrixes

The sequestering properties of CD-based materials can also be exploited to remove contaminants and toxins. In an interesting study, insoluble β-CD bead polymers (BBP) were tested in the removal of zearalenone (ZEN), a Fusarium-derived mycotoxin that exerts xenoestrogenic effects in animals and humans and is formed in cereals and cereal-based products [86]. The results showed that even relatively small amounts of BBP can strongly decrease the mycotoxin content of aqueous solutions (including beer), and they can be easily recycled with an EtOH/water (50:50) solution. In another study, alternariol (AOH), a mycotoxin that occurs in wine and tomato products as a contaminant, was removed by BBP from aqueous solutions (pH 3.0–7.4). BBP strongly decreased the AOH content of both wine and tomato juice samples, suggesting the suitability of CD polymers as AOH binders in some beverages [87].

Moreover, a study on the use of CD-NSs for removing organic toxic molecules from the body was recently published [88]. Different nanosponges were tested for the ability to complex indole, a metabolite of tryptophan formed by the gut microbiota which can form dangerous uremic toxins, such as indoxyl sulfate, which is metabolized from indole in the liver. Three of the four nanosponges tested were able to adsorb indole from aqueous solutions as well as from simulated gastric fluid. CD-NSs do not tend to accumulate or damage gastrointestinal tissues and are excreted from the GI tract with minimal absorption.

### 3.3. Role in the Extraction of Food-Related Compounds

Green extraction procedures are good alternatives to classical organic phase separative methods. In this context, CDs are interesting agents to consider due to the hydrophobic nature of bioactive compounds [129], not only for the green chemistry process but also for the additional protection of the bioactive compound [130]. Several authors have reported interesting studies with CD-based materials. The first application was to debitter grape fruit juice by removing naringin and limonin with the use of CDs crosslinked with epichlorohydrin, hexamethylene diisocyanate and phenyl isocyanate [89].

Curiously, a way to improve the application of CD-based materials is to optimize their recovery. Magnetic polymers are an attractive strategy because the recovery is easy. In this respect, carboxymethyl-hydroxypropyl-β-cyclodextrin polymer modified magnetic particles Fe_3_O_4_ (CM-HP-β-CDCP-MNPs) were recently prepared and applied to magnetic solid-phase extraction of rutin [90] with a maximum adsorption capacity of 67.0 mg g^−1^ for rutin with the equilibrium time of 30 min at room temperature. The polymer was able to be reused 10 times.

### 3.4. Effect on Flavor and Fragrances

Essential oils and fragrances are natural objectives for CD complexation, and many different complexes have been studied in the last few years [131,132]. In this respect, CD-based materials present several applications: Electrospun fibers of polystyrene containing volatile fragrance/flavor were facilitated by cyclodextrin inclusion complexes. Using menthol as a model, the complexes with α-, β- and γ-CD were prepared, and by adding polystyrene and electrospinning, the fibers were formed. The results showed that γ-CD was more effective for the stabilization and release of menthol at a broad temperature range (100–350 °C) when compared to α-CD and β-CD. The same strategy was applied to complex aniline and benzene in HPβ-CD and HPγ-CD fibers [133]. However, these types of studies are mainly focused on the use of CD monomers [132].

### 3.5. Cyclodextrin-Based Materials and Antimicrobials—Uses in Food Packaging

Cyclodextrin-based polymers have a potential role in the design of active packaging capable of increasing the shelf life and safety of foods while maintaining their quality. These polymers can be part of packaging films, laminates or containers made with a wide variety of natural or synthetic materials [61]. Additionally, the presence of cyclodextrins enhances the barrier properties of the packages and reduces residual organic volatile contaminants in the packaging materials [19]. Among them, those that incorporate antimicrobial or antioxidant agents that inhibit the growth of microorganisms that cause food spoilage or prevent the oxidation of food components stand out [2]. These bioactive compounds, which act as preservatives, can be kept in the polymer and released in a controlled way by the increase in humidity in the headspace of the packages, gradually exerting a beneficial effect on foods [134].

Current literature shows many cases that encapsulate the antimicrobial compound carvacrol in polymers of β-cyclodextrins with low-density polyethylene [91], chitosan [92], cellulose [93] and other materials. There are even examples of cardboard boxes that have a coating of carvacrol and essential oils complexed with β-cyclodextrin and halloysite nanotubes that improve the quality of stored fresh fruits [94], as well as TEMPO-oxidized cellulose nanocrystals with hydroxypropyl-β-cyclodextrin that have been used in food films in order to load and release molecules with antimicrobial and antioxidant properties (such as carvacrol and curcumin) [95]. Other authors developed titanium dioxide nanoparticles with α- and β-cyclodextrins as packaging materials that can embed common preservatives such as sorbic acid or benzoic acid [96]. Cyclodextrin nanosponges are gaining interest in this regard due to their modulable and multitasking capacities. It has been described that essential oil inclusion complexes in α-, β- and hydroxypropyl-l-β-cyclodextrin nanosponges could provide a controlled release of the antimicrobial agent in food packages while preserving the activity of the compound [97,98]. Moreover, since natural cyclodextrins are approved as food additives, there are some studies of edible coatings for fresh-cut fruits which integrate a bioactive compound complexed in polymers of β-cyclodextrin with natural polysaccharides such as chitosan or sodium alginate [99,100].

Another application in food packaging is the control of ripening in vegetables and fruits. Cyclodextrin-based polymers made of α- or γ-cyclodextrin with polystyrene [101,102] or metal–organic frameworks [103] have been shown to trap ripening agents such as ethylene, 1-methylcyclopropene or hexanal, resulting in modulation of the ripening process.

Besides the preformed inclusion complexes, the packaging material may instead contain “empty cyclodextrins” (without a complexed bioactive compound) capable of encapsulating molecules from the inside or outside of the package [2]. “Empty cyclodextrins” have been successfully grafted onto polyvinyl chloride to minimize plasticizer migration [104], onto zein copolymer to adsorb cholesterol [105] and onto low-density polyethylene to capture sulfur off-flavors [106].

The extrusion of mono- or multicomponent laminates is the most frequent technique for manufacturing food packaging. However, electrospinning is emerging as a promising methodology to create nanofibers with high nanoporosity, high surface/volume ratio and safety [135]. “Empty” or “filled” cyclodextrin polymers can be embedded into these electrospun nanofibers, improving the stability and slow release of bioactive compounds and the shelf life of foods. Some antimicrobial, antioxidant or flavoring agents such as allyl isothiocyanate [107], trans-cinnamaldehyde [108], essential oils [109,110,111], quercetin [112], ferulic acid [113], geraniol [114] or D-limonene [115] have been incorporated by this technique into polymers of β-, γ- or hydroxypropyl-β-cyclodextrin with polyethylene oxide (with soy protein), polylactic acid, zein, polyvinyl alcohol, chitosan, gliadin or pullulan. In addition, the electrospinning process does not prevent the development of edible packaging if biopolymers such as chitosan, cellulose, pullulan or zein are used in the matrix.

It is highlighted that in some cases the use of cyclodextrins-based polymers is the only way to retain and stabilize thermolabile bioactive compounds in the packaging material [136]. This is the case with 2-phenyl ethanol and geraniol, both fragrant agents with antimicrobial properties that are mostly volatilized and lost during packaging preparation. In contrast, the presence of polymers made of β-cyclodextrin with chitosan and γ-cyclodextrin with polyvinyl alcohol was shown to keep 90% or more of 2-phenyl ethanol and geraniol, respectively [114,116].

### 3.6. Cyclodextrin-Based Materials as Sensors for Food Safety and Health

As far as pollutants in food and water are concerned, volatile organic compounds (VOCs) are considered to be among the most present and dangerous substances [137,138] as they are able to permeate through polymeric films and vessels that are used as packaging material [139]. Moreover, the packaging itself, if not produced and used properly, can be an important source of contamination for food and beverages [140,141,142,143]. Halogenated organic compounds (HOCs) are another class of food pollutants of great concern for human health [144], as are nitroarenes [145] and pharmaceutical compounds [146]. Thus, the determination of such analytes is crucial in order to assess food and water safety.

Chemical sensing based on CD polymers is considered nowadays as a promising field as the need for fast, cheap and portable analytic detectors for the quantification of food and water pollutants has grown in recent years. The reasons behind this increasing interest are different. First, it has already been discussed how CDs are extremely versatile molecules since their hydrophobic inner cavity is particularly suitable to bind organic, inorganic and biological compounds to form host–guest complexes. The selectivity of this binding process highly depends on the size of the cavity and has a direct effect on the performance of the sensor. Second, CD polymers are known to be environmentally friendly compounds; they are relatively cheap to produce, and they can be synthesized by means of green methods [147,148,149]. Finally, the sensitivity, selectivity, dispersibility and the limits of detection of such sensors can be dramatically increased and finely tuned by modifying the concentration of CDs anchored to the polymer chains as a pendant group, or by using them directly as monomers [33,147,148,149,150,151].

CD-based sensors are usually classified based on the nature of the physical properties used to detect the analyte, even in food samples [33,152]. In this regard, it is shown that CD-based polymer sensors are highly selective for several health-harmful analytes; they can detect toxic or undesired chemicals even in presence of strong matrix effect generally with good limits of detectability, and the cost and difficulty of use of these sensors are normally low, making them remarkably suitable for applications in the food industry. Accordingly, several notable examples of CD-based polymer sensors capable of detecting the abovementioned classes of food and water pollutants in aqueous and nonaqueous environments are here presented and discussed along with some hints on future perspectives.

#### 3.6.1. Optical Sensors

It is known that the chemical environment deeply affects the optical properties of chromophores. When a fluorescent molecule is included in the cavity of a CD, a change in the color or in the intensity of fluorescence is expected. Therefore, these modifications in its photophysical behavior can be used as an analytic signal for its detection. In this case, the analyte produces the signal and the only function of the CD moieties is to selectively bind and retain the target. In 2006, Wang et al. [117] proposed a bifurcated optical fiber chemical sensor based on a β-CD polymer for the continuous quantification of bisphenol-A (BPA). They noticed a substantial enhancement of the intrinsic fluorescence of BPA recorded at 286 nm (excitation) and 312 nm (emission) when the inclusion complex is formed. The CD polymer was immobilized in a plasticized 5 µm thick PVC membrane, and the light was brought to the analytic flow-cell by means of two bifurcated fiber optic cables, one for the excitation and the other for the collection of emitted light. With this configuration, the sensor was reported to have a detection limit of 1.0 × 10^−6^ mol/L and a dynamic detection range from 6.0 × 10^−6^ to 1.0 × 10^−3^ mol/L.

Enhancement of the selectivity towards specific targets can be achieved by the use of molecular imprinting (MI) techniques [153]. When a crosslinked polymer is synthesized in presence of a template molecule, specific microcavities reminiscent of the shape of the template will form within the three-dimensional polymeric network. Once the template is removed, these pockets exhibit high binding affinity and selectivity towards the target. Molecularly imprinted β-CD polymers were immediately found to be effective for the separation of organic compounds [154,155]; however, they also attracted interest for sensing applications. Accordingly, Muk et al. [118] proposed an optical detector based on molecularly imprinted β-CD for the continuous quantification of the N-phenyl-1-naphtylamine (NPN) fluorescent dye. The polymer was synthesized using β-CD as monomer and 2,4-diisocyanate (TDI) as crosslinker in presence of NPN. A limit of detection of 1.38 µmol/L was found together with an analytical dynamic range up to 2.0 × 10^−3^ mol/L. However, probably the most remarkable result was that the molecularly imprinted CD-polymer sensor showed a sensitivity towards the analyte about the 16% higher than that of the non-molecularly imprinted control polymer.

The situation becomes more complex for analytes that do not show intrinsic fluorescence. In this case, specific chromophores can be appended to by means of more or less extended and flexible spacer chains. Accordingly, two types of fluorosensors can be identified, namely the “turn-off” and “turn-on” configurations.

CD chromophore-appended polymers can also show other peculiar behaviors. In 2006, Ogoshi et al. [119] synthesized a poly(phenylene-ethylene) flanked by two β-CD groups that exhibits blue fluorescence in DMF, while in aqueous media the polymer was found to be not completely soluble and to form partially quenched aggregates which led to green fluorescence. When 1-adamantanecarboxylic acid (AdCA) was added to the aqueous polymer, the green fluorescence at 490 nm shifted to deep blue at 460 nm with the increase in AdCA concentration. This is because the addition of the aliphatic guest, which is included in the cavities of the pending CD groups, dissociates the intermolecular aggregates and the green emission derived from the stacking of π-conjugated backbones is hence quenched. Another peculiar property is that shown by the helical stereoregular polyphenylacetylene with β-CD as pendant group polymer synthesized first in 2001 by Yashima et al. [120,156]. When 1-phenylethylamine was progressively introduced as guest molecule, the pitch of the helicity of the polymer was observed to change with consequent shift of the fluorescence emission from yellow to red. This change of color was observed only upon addition of the S enantiomer, while the R enantiomer did not induce color transitions.

As far as “turn-off” sensors are concerned, Ncube et al. [121] synthesized an azo-dye modified water-soluble CD polymer for the determination of chlorinated phenols in drinking water. The formation of host–guest inclusions with 4-chlorophenol and 2,4-dichlorophenol was proved to quench the fluorescence of the sensor proportionally to their concentration. The polymer was reported to have progressively higher sensitivity factors as the number of Cl substituents increased in pollutant molecules. Danquah et al. [122] prepared a highly porous crosslinked CD fluorescent polymer for the quantification of trinitrophenol and nitrobenzene. The inclusion of the analytes in the CD unit was observed to result in quenching of fluorescence up to 50%. This analytic method was reported to be cheap and suitable for rapid and in situ detection of explosive substances in water.

Because of their higher level of complexity, CD-polymer-based “turn-on” sensors are by far less studied and present in the literature; nevertheless, a few interesting examples are worth mentioning. Shi et al. [123] prepared a water-soluble crosslinked polymer composed of β-CD functionalized with tetrafluoroterephthalonitrile (TFT) for the quantification of the pharmaceutical product captopril in water and urine. Interestingly, the fluorescence of the probe was quenched when ferric ions were introduced. The sensor also exhibited a low detection limit of 1.8 × 10^−7^ mol/L and a linearity range between 9.2 × 10^−7^ mol/L and 4.6 × 10^−4^ mol/L. Interference experiments were conducted by introducing in the samples several species commonly present in urine such as amino acids, urea, glucose and inorganic ions; nevertheless, only the thiol-containing species such as cysteine interfered with the detection of captopril to a small extent.

#### 3.6.2. Other CD-Based Sensors

Besides optical and electrochemical sensors, CD-polymer detectors with functioning based on other chemical–physical principles are present in the literature. Additionally, the advance of nanomaterials such as carbon nanotubes and graphene opened new horizons in this field of applications along with supramolecular systems and CD–nanoparticle hybrids [33]. One interesting nonoptical and non-electrochemical sensor is the crosslinked b-CD/maleic acid polymer synthesized by Ju et al. [124] for the detection of benzene, toluene and p-xylene by means of quartz crystal microbalance sensing (QCM). QCM is a very well known and deeply studied piezoelectric mass transducer capable of detecting mass changes even smaller than nanograms and can be used for the quantification of volatile organic compounds [157]. This QCM-based detector showed linear ranges of 400, 300 and 150 ppm for benzene, toluene and p-xylene, respectively, with a higher sensitivity for the last compound. As far as nanomaterial-based CD sensors are concerned, both optical and electrochemical systems where CD is linked to carbon nanotubes [158,159,160,161], graphene [162,163] or nanoparticle hybrids [164] or is arranged in supramolecular structures [165,166] are well represented in the literature. These systems can occasionally guarantee better sensitivities than CD polymers. However, it has to be considered that their synthesis and their optimization processes are often much more complicated and expensive; consequently, the research on CD polymers is still a hot topic since they combine good sensitivity and selectivity with low costs and ease of preparation.

## 4. Critical Overview of the Capacity, Toxicity and Current Status of CD-Based Materials

Besides the potential activities of the CD-based materials in the field of food science, the industry currently prefers the use of natural members of the family. α-, β- and γ-CD are the approved members that can be used in the food industry [1,2] without significant toxicity due to their low capacity to be absorbed and fast degradation [167]. However, several of the studies reported showed a higher increase in concentration with CD-based polymers when compared to CD monomers, such as the case of KYNA [62], where the solubility increased 6.7-fold between CD monomers and polymers. This increase in solubility has been reported as an eventual increase in bioavailability [10]. For future uses, the degradation of bonds of CD-based polymers should be considered. Natural cross-linkers (e.g., citric acid) might be used for food administration as they present a nontoxic profile [88,168]. However, the use of chemical cross-linkers such as epichlorohydrin has been studied. In these cases, an exhaustive study must be carried out before administration.

The different degrees of substitution and even the nature of the linker can modulate the activity or toxicity of CD-based polymers, complexing some derivatives better than others. Recently, Kiss et al. [169] reported that methyl-β-derivatives provided better solubilization (and complexation) of cholesterol, with an optimum degree of substitution of 14. Although other derivatives such as HPβ-CD presented a lower capacity to solubilize cholesterol, significant differences were found between the cytotoxicity of highly toxic derivatives of methylated compounds (IC50: ≈50 mM, except those with a low degree of substitution) and other CDs.

Only the molecules approved as food additives can be used by the industry, limiting the total number of derivatives available in market applications. Nevertheless, and as this review shows, there are many research groups trying to generate new materials in view of a future application, and more and more are taking care of necessary aspects such as the toxicity and composition of their derivatives. For this reason, and although we have seen better results in increasing the apparent solubility of bioactive compounds or in food packaging, the most promising applications continue to be those related to the elimination of contaminants or sensors, where there is no contact with humans. To extend the range of applications and to achieve food additive approval, several studies about the toxicity of each derivative are necessary.

## 5. Conclusions

CD monomers have been used for several years in the food industry with good results. However, in the last few years, novel materials have been used in an effort to improve their capacities in different areas. In this review, a survey of the different CD-based materials was carried out, categorizing them into different groups and pointing out that the techniques used for CD monomers can generally be used with their derivatives too.

The derivatives were studied in the extraction of cholesterol, removal of contaminants and extraction of bioactive compounds; for their effects on flavor and fragrances; and as components in food packaging and novel sensors. In general, the capacity to modulate the cavity environment, increasing the selectivity, and the possibility to easily recover these materials encourage different research groups to follow this line of study, even if more studies about their toxicity are necessary to obtain the approval of international food safety organizations. 

As an evident prospect from this review, the versatility of the derivatives is wide and promising. The continuous research in the field and the cost reduction of this technology will promote the use of cyclodextrin-based polymers in the following years.

## Data Availability

Not applicable.
